# Differential SPARC mRNA expression in Barrett's oesophagus

**DOI:** 10.1038/sj.bjc.6601324

**Published:** 2003-10-14

**Authors:** J Brabender, R V Lord, R Metzger, J Park, D Salonga, K D Danenberg, P V Danenberg, A H Hölscher, P M Schneider

**Affiliations:** 1 Department of Visceral- and Vascular Surgery, University of Cologne, Joseph-Stelzmann Str. 9, Cologne 50931, Germany; 2 Department of Surgery, University of Southern California Keck School of Medicine and USC/Kenneth Norris Comprehensive Cancer Center, Los Angeles, CA 90033, USA; 3 Department of Biochemistry and Molecular Biology, University of Southern California Keck School of Medicine and USC/Kenneth Norris Comprehensive Cancer Center, Los Angeles, CA 90033, USA; 4 Response Genetics Inc., Los Angeles, CA 90033, USA

**Keywords:** Barrett's oesophagus, SPARC, molecular markers, oesophageal adenocarcinoma, gene expression, tumour biology

## Abstract

Barrett's oesophagus (BE) is the precursor lesion to adenocarcinoma of the oesophagus. Understanding of the molecular alterations in this multistage process may contribute to improved diagnosis and treatment. Secreted protein acidic and rich in cysteine (SPARC) is a matricellular protein that modulates cell adhesion and growth. Alterations in SPARC expression have been observed in a variety of solid tumours. The aim of this study was to assess the prevalence and timing of SPARC mRNA expression in Barrett's multistage disease and to investigate the impact of SPARC alterations on the development and progression of this disease. SPARC mRNA expression was measured using a quantitative real-time RT–PCR method in 108 specimens from 19 patients with BE without carcinoma, 20 patients with Barrett's-associated adenocarcinoma (EA), and a control group (CG) of 10 patients without evidence of gastro-oesophageal reflux disease. The median SPARC mRNA expression was significantly upregulated in BE tissues compared to paired normal oesophagus (NE) tissues for the BE group (*P*=0.004) and for the EA group (*P*<0.001). The SPARC mRNA expression was significantly higher in adenocarcinoma of the oesophagus compared to matching NE tissue and compared to Barrett's tissues in the EA group (*P*<0.001). Furthermore, SPARC expression values were significantly different between metaplastic and dysplastic Barrett's tissues (*P*=0.014). In histologically normal squamous oesophagus tissues obtained from carcinoma patients (EA group), the SPARC mRNA expression was significantly higher compared to NE mucosa from the BE group and the CG group (*P*=0.03). These findings suggest that the upregulation of SPARC mRNA expression is an early event in the development and progression of BE and EA, and that high SPARC expression may be a clinically useful biomarker for the detection of occult adenocarcinoma, and that a widespread ‘field effect’ is present in the NE of patients with oesophageal adenocarcinoma.

Barrett's oesophagus (BE), the replacement of the normal stratified squamous epithelium of the oesophagus by a metaplastic columnar lining, is a premalignant condition caused by chronic gastro-oesophageal reflux. This condition predisposes to the development of oesophageal adenocarcinoma, the incidence of which has been increasing rapidly in the United States and other Western countries (Bollschweiler *et al*, 2001). Oesophageal adenocarcinoma usually presents at an advanced stage and undergoes a rapidly fatal course, with 5-year survival rates of approximately 25–30% (Holscher *et al*, 1995). It is hoped that the identification of novel biomarkers associated with each Barrett's stage and with an increased cancer risk will lead to earlier detection and improved survival for patients with this disease.

Secreted protein acidic and rich in cysteine (SPARC), also known as BM-40 and osteonectin, is a developmentally regulated glycoprotein that is secreted into the extracellular matrix (ECM) (Bradshaw and Sage, 2001). It functions as a counteradhesive protein, modifying cell shape through the dissociation of focal adhesion, and modulates cell–matrix interactions by binding to the ECM (Bradshaw and Sage, 2001). Therefore, SPARC is thought to influence several biological processes, including differentiation, migration, and proliferation. Early in development, SPARC is expressed in tissues undergoing remodelling (Mundlos *et al*, 1992) and during vascular morphogenesis (Bradshaw and Sage, 2001). In the adult, it becomes more restricted, but can be activated in pathologic situations like wound healing (Reed *et al*, 1993). However, recent evidence suggests that alterations in SPARC expression are common in various human malignancies, including melanoma (Ledda *et al*, 1997b), glioma (Schultz *et al*, 2002), invasive meningioma (Rempel *et al*, 1999), hepatocellular carcinoma (Le Bail *et al*, 1999), colon (Porte *et al*, 1995), breast (Gilles *et al*, 1998), and prostate (Thomas *et al*, 2000) cancers. To date, however, there are no detailed studies on SPARC expression in BE and Barrett's-associated adenocarcinoma (EA) of the oesophagus. The aim of the underlying study was to analyse SPARC mRNA expression in the development and progression of BE and EA, and to determine the potential of SPARC mRNA quantitation in the clinical management of this disease.

## MATERIAL AND METHODS

### Tissue samples for RT–PCR

A total of 108 tissue samples obtained at endoscopy and operation from 19 patients with BE without adenocarcinoma (BE group), 20 patients with EA (EA group), and 10 patients with no symptomatic, endoscopic, or histopathologic evidence of BE or chronic gastro-oesophageal reflux disease (control group, CG) were collected and immediately frozen in liquid nitrogen. There were 31 men and 18 women, with a median age of 60.1 years (range 24–76 years). Endoscopic biopsies were obtained according to a protocol that required biopsy at 2 cm intervals from each quadrant (anterior, posterior, right, and left lateral positions) of the visible length of Barrett's mucosa and an additional biopsy from the normal appearing squamous mucosa of the oesophagus. Normal oesophagus (NE) biopsies were taken at least 4 cm proximal to the macroscopically abnormal epithelium. Part of the specimen or an adjacent specimen was fixed in formalin and embedded in paraffin for histopathological examination.

Specimens were classified as intestinal metaplasia (IM) if IM, but no dysplasia or cancer was present. Specimens were classified as dysplastic if either low-grade dysplasia (LGD) or high-grade dysplasia (HGD) was present. Dysplastic tissues were not divided into LGD or HGD groups because areas of LGD and HGD were commonly present in the same specimen. Using these criteria, the following tissues were analysed for SPARC mRNA expression: Barrett's IM (*n*=16), Barrett's dysplasia (*n*=3), and matching normal squamous tissue (*n*=19) in the BE group, Barrett's adenocarcinoma of the oesophagus (*n*=20), Barrett's IM (*n*=5), Barrett's dysplasia (15), and matching normal squamous oesophagus tissues (*n*=20) in the EA group, and normal squamous oesophagus tissues (*n*=10) in the CG, for a total of 108 specimens.

### RNA extraction and cDNA synthesis

Total RNA was isolated by a single-step guanidinium isothiocyanate method using the QuickPrep™*Micro* mRNA Purification Kit (Amersham Pharmacia Biotech Inc., Piscataway, NJ, USA) according to the manufacturer's instructions, and cDNAs were prepared as previously described (Chomczynski and Sacchi, 1987; Lord *et al*, 2000).

### PCR quantification of mRNA expression

Quantitation of SPARC cDNA and an internal reference cDNA (*β*-actin) was carried out using a fluorescence detection method (ABI PRISM 7700 Sequence Detection System, (TaqMan®) Perkin-Elmer (PE) Applied Biosystems, Foster City, CA, USA), as described (Gibson *et al*, 1996; Heid *et al*, 1996; Lord *et al*, 2000).

The PCR reaction mixture consisted of 600 nM of each primer, 200 nM probe, 5 U AmpliTaq Gold Polymerase, 200 *μ*M each dATP, dCTP, dGTP, 400 *μ*M dUTP, 5.5 mM MgCl_2_, and 1 × Taqman Buffer A containing a reference dye, to a final volume of 25 *μ*l (all reagents Perkin-Elmer (PE) Applied Biosystems, Foster City, CA, USA). Cycling conditions were 50°C for 10 s, 95°C for 10 min, followed by 46 cycles at 95°C for 15 s and 60°C for 1 min. The primers and probes used are listed in Table 1

**Table 1:** PCR primers and probes

GenBank accession: NM_003118 SPARC
Forward primer: SPARC
Sequence: 5′-TCTTCCCTGTACACTGGCAGTTC-3′
Reverse primer: SPARC
Sequence: 5′-AGCTCGGTGTGGGAGAGGTA-3′
TaqMan probe: SPARC
Sequence: 6FAM 5′-CAGCTGGACCAGCACCCCATTGAC-3′TAMRA
GenBank accession: BC016045 *β*-actin
Forward primer: *β*-actin
Sequence: 5′-TGAGCGCGGCTAC AGCTT-3′
Reverse primer: *β*-actin
Sequence: 5′-TCCTTAATGTCACGCACGATTT-3′
TaqMan probe: *β*-actin
Sequence: 6FAM5′-ACCACCACGGCC GAGCGG-3′TAMRA

. The set of SPARC primers was designed to be RNA specific by spanning exons 8 and 9.

### Statistical analysis

Taqman® analyses yield values that are expressed as ratios between two absolute measurements (gene of interest/internal reference gene). SPARC expression levels in adenocarcinoma, BE, and normal squamous oesophagus tissues were compared using the Kruskal–Wallis test to identify significant differences in the expressions among the histopathologic groups. The Kruskal–Wallis test was also used to compare the three groups of NE tissues. When the overall Kruskal–Wallis test (comparing three groups) was significant at the 0.05 level, pairwise comparisons were based on the Mann–Whitney test and the nominal *P*-value was reported. The Wilcoxon signed-rank test was used for comparison of paired tissues. Statistical significance (with two-sided tests) was set at the 0.05 level.

## RESULTS

SPARC mRNA expression was detectable by quantitative real-time PCR (Taqman®) in all 108 (100%) specimens. Analysed according to histopathologic group, the median SPARC mRNA expression was lowest in normal squamous oesophagus tissues (median 0.95, range 0.14–16.50), intermediate in BE (median 3.89, range 0.09–53.62), and highest in EA of the oesophagus (median 13.79, range 1.81–105.22; *P*<0.001, Kruskal–Wallis test).

**Figure 1:**
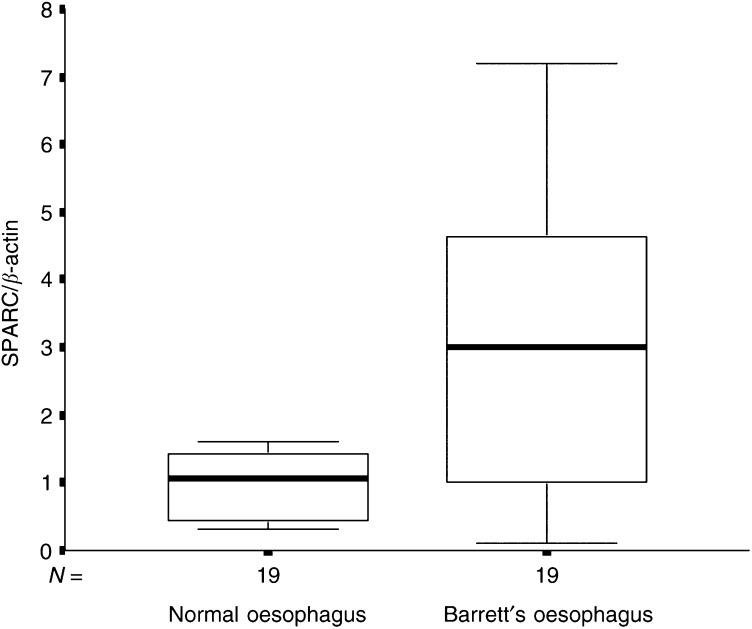
Box and whisker plots of relative SPARC mRNA expression levels for NE and BE tissues from patients with the maximum diagnosis of BE. The boxes show the 25th and 75th percentile (interquartile) ranges. Median values are shown as a horizontal black bar within each box. The whiskers show levels outside the 25th and 75th percentiles (*P*=0.004).

Of the 19 (73.7%) patients, 14 with the maximum diagnosis of BE (BE group, *n*=19) had higher SPARC mRNA expression levels in Barrett's epithelium compared to matching normal squamous oesophagus tissues. The median SPARC mRNA expression in normal squamous oesophagus tissues was 1.07 (range 0.29–2.94) and 2.99 in BE (range 0.09–7.19; *P*=0.004, Wilcoxon's test; Figure 1

; Table 2

**Table 2:** SPARC mRNA expression in tissues from patients with adenocarcinoma and BE

		**SPARC expression**			
**Pathology**	***n***	**Median**	**Range**	**Interquartile range (25th–75th percentiles)**	***P*-value**
*EA group*	20				
Adenocarcinoma		13.79	1.8–105.2	7.79–37.18	<0.001
BE		6.91	0.1–53.62	3.67–13.69	
NE		1.79	0.1–16.5	0.59–3.05	
					
*BE group*	19				
BE		2.99	0.09–7.19	0.99–4.97	0.004
NE		1.07	0.29–2.94	0.42–1.52	
*CG group*	10				
NE		0.58	0.45–1.17	0.51–0.63	

EA=adenocarcinoma group; BE=Barrett's oesophagus group; CG=control group.

).

**Figure 2:**
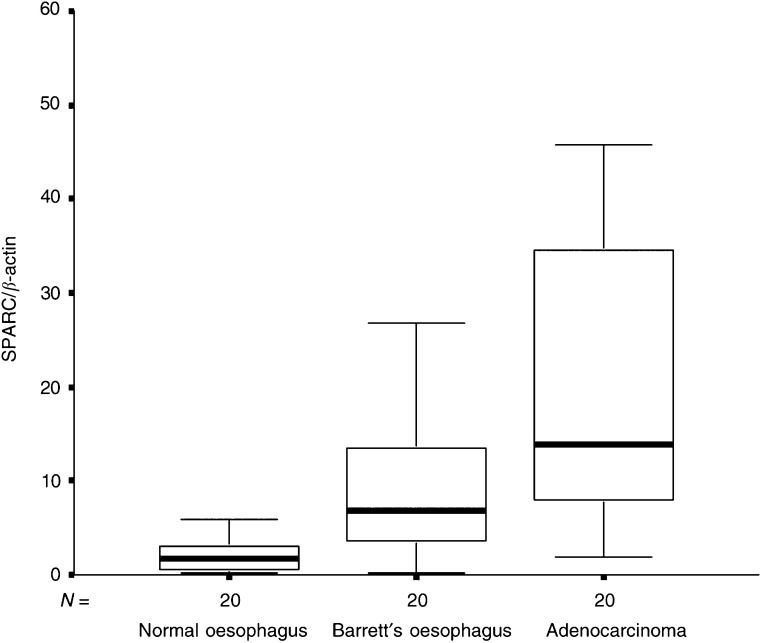
Box and whisker plots of relative SPARC mRNA expression levels for NE, BE, and EA tissues from patients with EA of the oesophagus. NE *vs* BE, *P*=0.001; NE *vs* EA *P*<0.001; BE *vs* NE, *P*=0.004.

In the group of patients with EA (EA group, *n*=20), 18 of 20 (90%) patients had higher SPARC mRNA expression levels in cancer tissues compared to matching NE tissues. The median SPARC mRNA expression was 1.79 (range 0.14–16.54) in NE, 6.91 (range 0.11–53.62) in Barrett's epithelium, and 13.79 (range 1.81–105.22) in EA (*P*<0.001, Kruskal–Wallis test). Table 2 and Figure 2

show that the median SPARC mRNA expression was significantly higher in EA compared to matching NE tissues and Barrett's epithelium.

**Figure 3:**
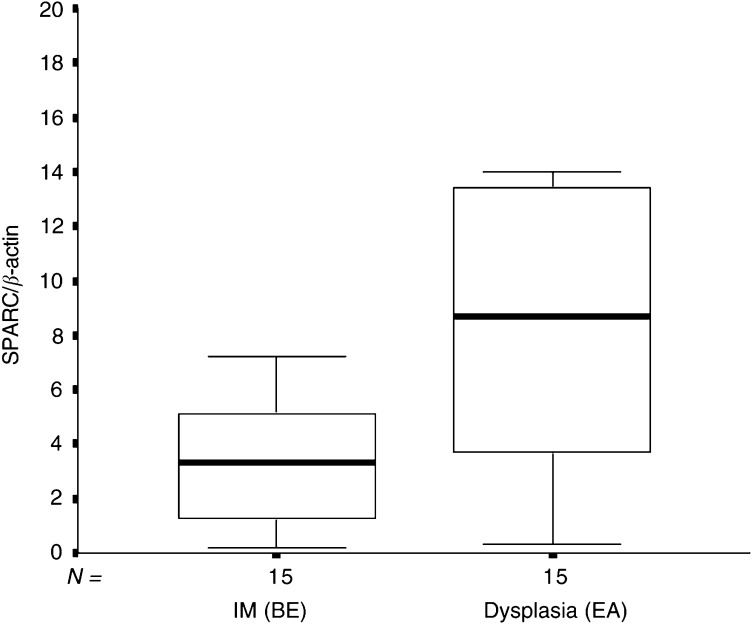
Box and whisker plots of relative SPARC mRNA expression for metaplastic oesophagus tissues from patients with BE (BE group) and dysplastic oesophagus tissues from patients with EA (EA group) (*P*=0.014).

To search for further differences in the SPARC mRNA expression between the different stages of Barrett's progressive disease, we compared the median SPARC expression of metaplastic oesophagus (IM) tissues from patients with the maximum diagnosis of BE (BE group) with dysplastic Barrett's tissues from patients with adenocarcinoma of the oesophagus (EA group). As shown in Figure 3

, the median SPARC expression was significantly lower in Barrett's IM (*n*=16; median 3.16, range 0.21–7.19) compared to Barrett's dysplasia (*n*=15; median 8.69; range 0.34–53.62; *P*=0.014; Mann–Whitney test).

**Figure 4:**
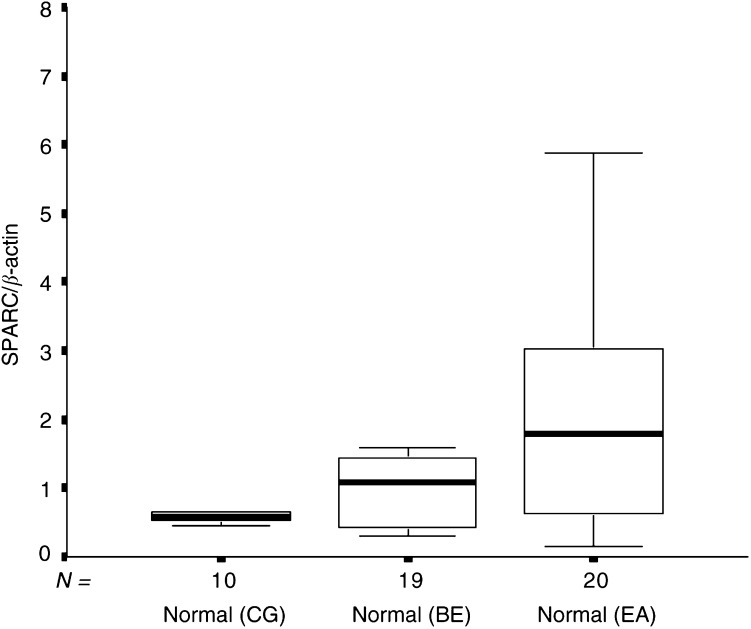
Box and whisker plots of relative SPARC mRNA expression levels for normal squamous oesophagus tissues from a CG without evidence of Barrett's oesophagus or chronic gastro-oesophageal reflux, and patients with BE (BE group), and patients with EA (EA group). CG *vs* BE, *P*=NS; CG *vs* EA, *P*=0.02; BE *vs* EA, *P*=0.03.

Overall, the three groups of NE tissue revealed substantial differences in SPARC expression levels (*P*=0.03, Kruskal–Wallis test). The median SPARC mRNA expression in the group of histologically normal squamous oesophagus tissues from patients with adenocarcinoma (median 1.79; range 0.14–16.54) was significantly higher than the median SPARC expression found in normal squamous oesophagus tissues from patients with BE only (median 1.07; range 0.30–2.94; *P*=0.04; Mann–Whitney test) and normal squamous oesophagus tissues obtained from the CG (median 0.58; range 0.45–1.17; *P*=0.02; Mann–Whitney test; Figure 4

).

## DISCUSSION

The main risk factor for the development of oesophageal adenocarcinoma is the presence of BE. The mechanisms underlying the increased cancer development in this tissue are not fully understood, but substantial evidence exists that progression to Barrett's cancer is associated with a variety of genetic and epigenetic alterations (Rabinovitch *et al*, 1989; Reid *et al*, 1996; Schneider *et al*, 1996). This study demonstrates that SPARC mRNA expression is upregulated in BE and EAs. SPARC expression was increased even in Barrett's IM tissues, indicating that the induction of the expression of this gene is an early event in the Barrett's adenocarcinoma progression. There was considerable variation of SPARC mRNA expression levels in tissues at each Barrett's stage, but the analysis of grouped results showed that there was a significant progressive elevation of SPARC expression through the stages of Barrett's IM to adenocarcinoma of the oesophagus. Our findings complement the results of previous studies that reported alterations in SPARC expression in various human cancers. The overexpression of SPARC has been detected in melanoma (Ledda *et al*, 1997a), colorectal cancer (Porte *et al*, 1995), breast cancer (Bellahcene and Castronovo, 1995), hepatocellular carcinoma (Le Bail *et al*, 1999), invasive meningiomas (Rempel *et al*, 1999), and prostate cancer (Thomas *et al*, 2000). Moreover, it has been reported that SPARC promotes cell migration and invasion in prostate cancer (Jacob *et al*, 1999) and glioblastoma (Golembieski *et al*, 1999). The suppression of SPARC expression by antisense RNA results in a significant decrease in the tumorigenicity of melanoma cells (Ledda *et al*, 1997b). These results suggest that inappropriate SPARC expression is a somewhat specific effect that contributes to tumour development and is not simply a function of generalised inflammation in BE.

Our results suggest that quantitation of SPARC mRNA expression offers promise as a biomarker for following disease progression in individuals with BE. It seems plausible that BE patients with a more abnormal SPARC expression profile are at greater of progression to higher disease stages due to increased capacity for invasion and proliferation, but this needs to be demonstrated in studies of sequential biopsies in individual patients. It is likely that molecular diagnosis and staging of BE will probably require the assessment of a panel of gene expressions. Studies from this institution and elsewhere suggest that many genes have significantly different expressions or mutation frequencies at different Barrett's stages (Schneider *et al*, 1996; Eads *et al*, 2000; Lord *et al*, 2000, 2001; Brabender *et al*, 2001a, 2001b, 2002)

The mechanism leading to inappropriate SPARC expression in tumorigenesis, and whether the effects of SPARC on invasion and proliferation are induced by mechanisms that are linked or mutually exclusive is not yet known, and was not purpose of this investigation. Further studies are warranted to determine the underlying mechanisms leading to altered SPARC expression in this disease.

SPARC mRNA expression levels were significantly higher in normal squamous oesophagus tissues from patients with cancer compared to patients with the maximum diagnosis of BE and the CG without the evidence of BE or chronic gastro-oesophageal reflux. We and others have found similar evidence for the presence of a widespread oncogenic ‘field effect’ in the NE of cancer patients in studies of gene expression and DNA methylation analysis (Eads *et al*, 2000; Lord *et al*, 2000; Brabender *et al*, 2001a, 2001b, 2002). One explanation for this field change is that due to an injurious environmental agent, for example, the gastro-oesophageal refluxate, some of the early events of tumorigenesis have already occurred. These early events might predispose the apparently normal squamous oesophagus tissue to undergo further genetic changes leading ultimately to the development of BE and adenocarcinoma. An alternative explanation is that clones of abnormal cells, in the presence of cancer, have expanded widely throughout the mucosa to replace previously normal cells. In either case, it is apparent that genetic changes can precede the appearance of morphologic changes in this disease.

In summary, these data suggest that upregulation of SPARC mRNA expression is an early event in Barrett's multistage disease, which already occurs at the level of IM and further increases during progression to cancer. The presence of oesophageal adenocarcinoma seems associated with an ‘oncogenic’ field effect on the normal squamous oesophagus mucosa. Quantitation of SPARC mRNA expression might be a novel biomarker for the detection of cancer in patients with BE.
